# Loss of *Pax6* Causes Regional Changes in *Dll1* Expression in Developing Cerebral Cortex

**DOI:** 10.3389/fncel.2019.00078

**Published:** 2019-03-06

**Authors:** Elena Dorà, David J. Price, John O. Mason

**Affiliations:** Centre for Discovery Brain Sciences, University of Edinburgh, Edinburgh, United Kingdom

**Keywords:** Pax6, cortical development, progenitor cells, delta-like 1, Notch pathway

## Abstract

The transcription factor Pax6 controls multiple aspects of forebrain development. Conditional deletion of *Pax6* in embryonic mouse cortex causes increased proliferation of cortical progenitor cells and a concomitant decrease in neural differentiation. Notch signaling regulates the balance between proliferation and differentiation of cortical progenitor cells, suggesting a possible connection between Pax6 and Notch signaling. We investigated how expression of the Notch ligand *delta-like 1* (*Dll1*) is altered by loss of Pax6. Acute cortex-specific deletion of Pax6 resulted in a widespread decrease in the density of *Dll1*+ cells at embryonic days 12.5 and 13.5 (E12.5 and E13.5). In constitutive loss-of-function mutants, decreases in the densities of *Dll1*+ cells were more limited both spatially and temporally. Controlled over-expression of Pax6 had no detectable effect on *Dll1* expression. The proneural transcription factor Neurog2 is a target of Pax6 that can activate *Dll1* expression and we found clear co-expression of *Neurog2* and *Dll1* in radial glial progenitors, suggesting that Pax6’s effect on *Dll1* could be mediated through Neurog2. However, we found no change in *Dll1*+ cells in *Neurog2*^−/−^ cortex suggesting either that Neurog2 is not directly involved, or that its loss of function in embryonic cortex can be compensated for.

## Introduction

As the brain develops during embryogenesis it must ensure that appropriate numbers of cells are born at the appropriate time, such that each region of the brain reaches the correct final size. To achieve this, the relative rates of proliferation and differentiation of neural progenitor cells must be tightly controlled. The earliest progenitor cells in the embryonic cortex, neuroepithelial cells, divide symmetrically to generate two new progenitors, expanding the progenitor pool. As development proceeds, neuroepithelial progenitors transform into radial glia cells (RGCs), a major class of neural progenitors. RGCs may divide symmetrically to give rise to two identical progenitor cell daughters, or asymmetrically, giving rise to one radial glial cell and one more differentiated cell—either a neuron, or an intermediate progenitor cell (IPC). In the mouse, most IPCs divide symmetrically to produce two neurons, but a small proportion divide asymmetrically to give rise to one progenitor and one neuron (Haubensak et al., 2004; Noctor et al., 2004; Pontious et al., 2008). Clearly, RGCs must carefully balance their output between neurogenic cells (IPCs and neurons) and new RGC progenitors. If too many neurogenic cells are produced early, this would lead to a reduction in progenitor numbers and an overall smaller cortex. Conversely, too few neurogenic divisions would increase progenitor numbers and cortical size.

The transcription factor Pax6 is a key regulator of forebrain development, with diverse roles in corticogenesis, including control of cortical cell number (reviewed by Manuel et al., 2015; Ypsilanti and Rubenstein, 2016). Pax6 is expressed highly in RGCs, but its levels decrease in IPCs (Elsen et al., 2018). We recently showed that acute deletion of Pax6 in the embryonic cortex leads to both an increase in cortical progenitor proliferation and a decrease in neural differentiation (Quintana-Urzainqui et al., 2018). This suggests that Pax6 can regulate the output from cortical RGCs by influencing the balance between their proliferation and differentiation. As a transcription factor, Pax6’s effects on RGC output are most likely exerted through control of target gene expression. Many Pax6 regulated genes have been identified (Sansom et al., 2009; Wolf et al., 2009; Mi et al., 2013; Xie et al., 2013; Sun et al., 2015; Quintana-Urzainqui et al., 2018) including the pro-neurogenic transcription factor Neurog2 (previously known as Ngn2), which is directly regulated by Pax6 (Scardigli et al., 2003). In addition to its pro-neurogenic activity, Neurog2 activates expression of the Notch ligand *Delta-like 1* (*Dll1*; Castro et al., 2006; Shimojo et al., 2008). Notch signaling is a well-characterized regulator of neural progenitor maintenance and differentiation. When Notch signaling is active, the Notch ligands Dll1 and Jagged engage Notch receptors on the surface of neighboring cells and induce expression of genes that inhibit neural differentiation, thereby maintaining the cell in a proliferative state (Pierfelice et al., 2011). Taken together, this suggests a plausible hypothesis to explain how Pax6 could influence behavior of the RGC progenitor pool in embryonic cortex. Pax6 activates Neurog2 expression in RGCs, in turn Neurog2 acts both to promote a pro-neural fate in cells expressing it and to drive expression of *Dll1*, which could then feed back to neighboring cells to promote their proliferation and thereby reduce their likelihood of becoming pro-neurogenic. Consistent with this idea, microarray and transcriptome sequencing studies have shown that overall levels of *Dll1* expression are significantly decreased in *Pax6*^−/−^ mutant cortex (Mi et al., 2013; Quintana-Urzainqui et al., 2018) and increased in transgenic mice that overexpress Pax6 (Sansom et al., 2009), but it is not known how the cellular pattern of *Dll1* expression is affected in the absence of Pax6. Pax6 is expressed in a gradient across the developing cortex and some of its effects on cell cycle have been shown to be confined to regions where Pax6 expression is highest (Mi et al., 2013). It is therefore important to examine the spatial effects of Pax6 loss on *Dll1* expression in more detail.

In the present study, we investigated how the spatial distribution of *Dll1*-expressing cells is affected by loss of Pax6. We found significantly reduced densities of *Dll1*-expressing cells in areas of the embryonic cortex in both constitutive and conditional* Pax6* mutants, consistent with the suggestion that Pax6 promotes *Dll1* expression. Interestingly, the effect on *Dll1* expression was greater in conditional *Pax6* mutants than in *Pax6*^−/−^ null mutants of the same age, suggesting the presence of a compensatory mechanism which has not had time to take effect in the conditional mutants. We also found that many RGCs co-expressed Neurog2 and *Dll1*, as predicted by our hypothesis. However, while loss of Pax6 led to a dramatic decrease in Neurog2 expression, its effect on *Dll1* expression was less pronounced and *Dll1* expression was unaffected in *Neurog2*^−/−^ mutant cortex, suggesting that additional levels of control of *Dll1* expression are present.

## Materials and Methods

### Mice

All animal experiments were conducted in accordance with the guidelines of the UK Animals (Scientific Procedures) Act 1986 and were approved by Edinburgh University’s Animal Ethics Committee. For all crosses, the morning on which the vaginal plug appeared was deemed embryonic day 0.5 (E0.5). *Pax6*^−/−^ embryos were obtained by crossing *Pax6*^Sey/+^ heterozygotes (Hill et al., 1991) and non-homozygous littermates were used as controls. Pax6 cortex-specific conditional mutant embryos (*Emx1cre-ER*^T2^;*Pax6*^loxP/*loxP*^, abbreviated to cKO) were obtained as described previously (Mi et al., 2013). Cre activity was induced by administering 10 mg of tamoxifen (Sigma) by oral gavage at E9.5. *PAX77* mice (Schedl et al., 1996) carry a yeast artificial chromosome transgene containing multiple copies of human *PAX6* under the control of its own regulatory elements and express PAX6 protein at approximately three-fold of normal levels (Manuel et al., 2007). *Neurog2*^−/−^ embryos were obtained by crossing *Neurog2*^cre/+^ heterozygotes in which insertion of cre coding sequence creates a null allele of *Neurog2* (Zirlinger et al., 2002). Embryos were dissected in cold phosphate buffered saline (PBS), fixed overnight in 4% paraformaldehyde at 4°C, cryo-protected overnight in 30% sucrose at 4°C, snap frozen in 50:50, OCT: 30% sucrose mix and cryosectioned at a thickness of 10 μm.

### *In situ* Hybridization/Immunohistochemistry

*In situ* hybridization was performed as previously described (Yu et al., 2009). The *Dll1* riboprobe (Ramos et al., 2010) was labeled with digoxigenin for both chromogenic and fluorescent *in situ* hybridizations. The *Neurog2* riboprobe (Gradwohl et al., 1996) was labeled with dinitrophenol for fluorescent *in situ* hybridizations. For double *in situ* hybridization, probes were detected sequentially and slides were incubated in 10 mM HCl prior to detection of the second probe.

For immunohistochemistry subsequent to fluorescent *in situ* hybridization, antigen retrieval was carried out in 10 mM citrate buffer pH 6.0 in a 90°C water bath for 40 min. The primary antibody was rabbit anti-Tbr2 (1:100; Abcam Ab23345) and the secondary antibody was goat anti-rabbit IgG Alexa Fluor^®^ 488 (1:400; Abcam). Nuclei were counterstained with DAPI (Thermo Fisher Scientific, Waltham, MA, USA).

### Cell Counting

The density of *Dll1*-expressing cells and total cell density were quantified using images produced from *in situ* hybridization and H&E staining experiments. Sample areas were defined at rostral, central and caudal regions of the cortex and cells were counted in both medial and lateral areas of the cortex at each region (Figure 2A schematic). Three sections per region, per embryo were analyzed for each mutant and the appropriate control littermate. Three embryos of each genotype and developmental stage were analyzed, except for analysis of *Dll1+* cell density in *PAX77* embryos at E12.5 where five embryos were used.

**Figure 1:**
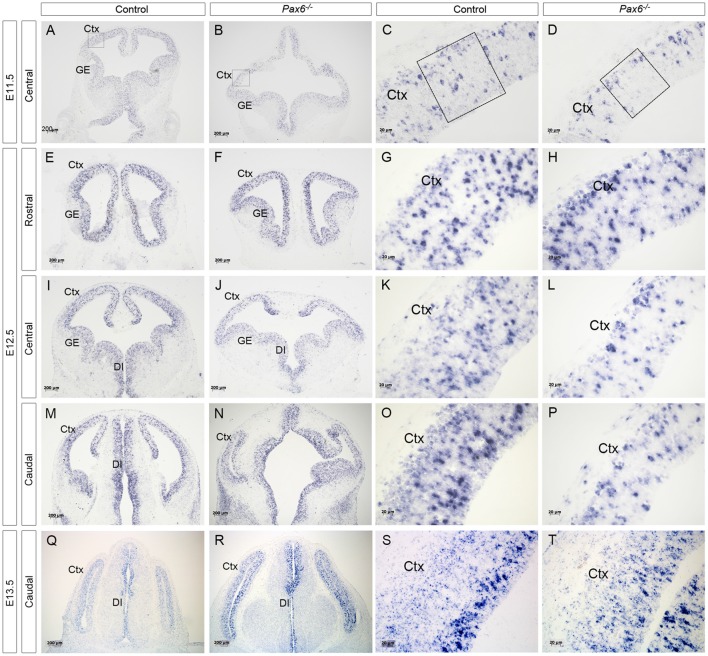
Expression of delta-like 1 (*Dll1*) in *Pax6*^−/−^ and control forebrain between E11.5 and E13.5. *In situ* hybridization showing *Dll1* expression in *Pax6*^−/−^ mutant and control forebrains at E11.5 **(A–D)**, E12.5 **(E–P)** and E13.5 **(Q–T)**. At E11.5, the diencephalon in *Pax6*^−/−^ mutants has a more open structure than in controls (Caballero et al., 2014) and the dorsal telencephalon is smaller, leading to the altered appearance of the section in **(B)**. Scale bars two leftmost columns = 200 μm. Scale bars in rightmost columns = 20 μm. Abbreviations: Ctx, cortex; GE, ganglionic eminence; DI, diencephalon. Boxes in **(C,D)** indicate representative areas used for counting *Dll1*+ cells.

**Figure 2:**
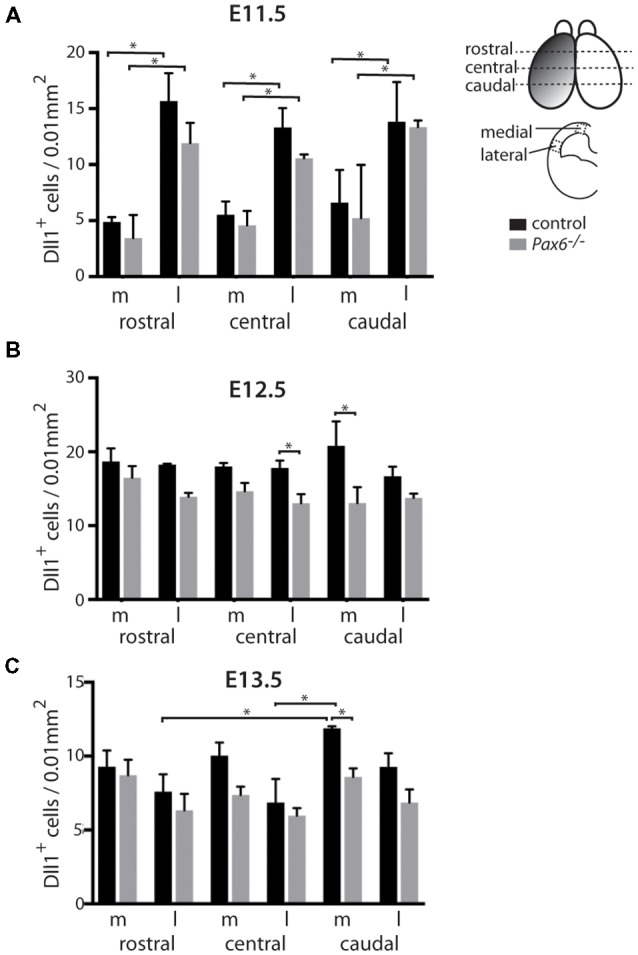
Quantitation of *Dll1+* cells in *Pax6*^−/−^ mutant and control cortex at E11.5, E12.5 and E13.5. Bar graphs showing the density of *Dll1*-expressing cells in specific regions of the cortex in *Pax6*^−/−^ mutant and control embryos at E11.5 **(A)** E12.5 **(B)** and E13.5 **(C)**. The schematic diagram in the top right corner indicates approximate positions from which samples were taken for counting. Shading on the left hemisphere indicates the gradient of Pax6 expression. *N* = 3 embryos of each genotype at each age. *p < 0.05, Tukey’s multiple comparison test.

The width of the cortex at each area counted was measured and a square was drawn on the tissue with each side equating to the recorded width, to create the sample area for counting. The total number of cells within each sample area was counted then divided by the area to give the cell density, expressed as number of cells per 0.01 mm^2^. The average cell density for each region was calculated and statistical analysis by two or three-way analyses of variance (ANOVA) and Tukey’s multiple comparison test was conducted.

Cell count analysis was carried out using ImageJ software and statistical analysis was carried out using Prism 6 software.

## Results

### Expression of *Dll1* in *Pax6*^−/−^ Mutant Embryonic Cortex

Previous analysis of the cortical transcriptome of *Pax6*^−/−^ mutant embryos revealed that overall *Dll1* expression is reduced compared to controls (Sansom et al., 2009). To determine how absence of Pax6 affects *Dll1* expression in the embryonic cortex in more detail, we carried out *in situ* hybridization for *Dll1* on *Pax6*^−/−^ mutant and control embryos between E11.5 and 13.5 (Figure 1). In controls, *Dll1* was expressed in a salt and pepper pattern throughout the telencephalic tissue at E11.5, E12.5 and E13.5, in agreement with previous studies (Lindsell et al., 1996; Kageyama et al., 2008; Kawaguchi et al., 2008; Shimojo et al., 2008; Yoon et al., 2008). The distinctive salt and pepper pattern is a consequence of periodic oscillations in *Dll1* mRNA levels, as found for a number of Notch pathway components (Shimojo et al., 2016). *Pax6*^−/−^ embryos showed a similar salt-and-pepper pattern of *Dll1* expression at all three ages (Figure 1). The density of Pax6-expressing cells appeared lower in some mutant sections (e.g., compare panels Figures 1D,L,P to Figures 1C,K,O). To test whether *Dll1* expression was altered in the *Pax6* mutant embryos, we systematically counted the density of *Dll1*-expressing cells in age-matched mutant and control embryos. Pax6 is expressed in a gradient across the cortex (rostrolateral high to caudomedial low) so, to test for possible differential effects caused by differing levels of Pax6 expression, we calculated the density of *Dll1-expressing* cells in both medial and lateral areas of sections taken from rostral, central and caudal levels of *Pax6*^−/−^ and control cortex at E11.5, 12.5 and 13.5 (Figure 2). At E11.5, the density of *Dll1*-expressing cells appeared consistently higher in lateral regions of the cortex than in equivalent medial regions, both in control and *Pax6*^−/−^ embryos (Figure 2A). Two-way ANOVA analysis confirmed a significant effect of region (*p* < 0.001) and *post hoc* analysis by Tukey’s multiple comparison test showed a significantly lower density of *Dll1-expressing* cells in medial than lateral cortex at rostral (*p* = 0.003), central (*p* = 0.015) and caudal (*p* = 0.03) levels in control embryos. A similar pattern was found in *Pax6*^−/−^ mutants and ANOVA showed that there were no significant differences between mutant and control cortices in any of the six counted areas.

At E12.5, ANOVA showed that there were no significant regional differences in the density of *Dll1*-expressing cells, either in controls or *Pax6*^−/−^ mutants (Figure 2B), indicating that the medial—lateral difference observed at E11.5 has disappeared by E12.5. *Dll1*-expressing cell density was lower in all regions of *Pax6*^−/−^ cortex compared to controls (Figure 2B) and ANOVA indicated a significant effect of genotype (*p* < 0.001). *Post hoc* analysis (using Tukey’s multiple comparison test) confirmed significant decreases in the density of *Dll1*-expressing cells in *Pax6*^−/−^ mutants in both centrolateral and caudomedial cortex (*p* = 0.028 and < 0.0001, respectively), but the decreases in other regions were not significant.

At E13.5, two-way ANOVA showed significant effects of both region and genotype (both *p* < 0.001). In control embryos, *Dll1*-expressing cell density was highest in caudomedial cortex and *post hoc* analysis (Tukey) showed that *Dll1*-expressing cell density in caudomedial cortex was significantly higher than in rostrolateral (*p* = 0.0006) and centrolateral cortex (*p* < 0.0001) but differences to other regions were not significant (Figure 2C). *Post hoc* analysis revealed that these regional differences were not present in *Pax6*^−/−^ mutants (Figure 2C). We found a significant decrease in *Dll1*-expressing cell density in caudomedial cortex of *Pax6*^−/−^ mutants when compared to the same region in controls (*p* = 0.012), but there was no significant difference between mutant and control embryos in any of the other regions tested (Figure 2C).

We found no significant differences in the overall cell density between control and mutant cortex at E11.5 (82.3 ± 6.8 vs. 89.7 ± 15.2 cells/0.01 mm^2^), E12.5 (101.3 ± 4.4 vs. 101 ± 8.8 cells/0.01 mm^2^) or E13.5 (111 ± 6.3 vs. 108 ± 8.1 cells/0.01 mm^2^). This indicates that the observed differences in the density of *Dll1*-expressing cells in *Pax6*^−/−^ cortex are due to changes in the number of *Dll1*-expressing cells, and not simply a consequence of a lower total number of cells in *Pax6*^−/−^ mutants.

These findings suggest that Pax6 loss lowers the proportions of *Dll1*-expression produced in caudomedial regions but leaves open the possibility that it also affects production in other area. To examine this further, we switched from constitutive to conditional mutants.

### Conditional Mutation of *Pax6* Causes a Widespread Decrease in *Dll1* Expression

**Figure 3:**
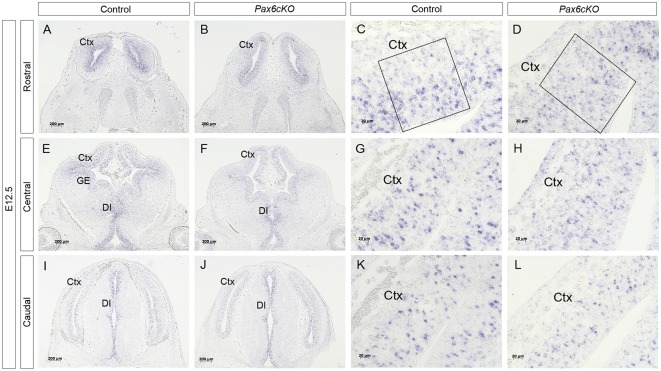
Expression of *Dll1* in *Pax6* cKO mutant forebrain at E12.5. *In situ* hybridization showing *Dll1* gene expression in rostral **(A–D)**, central **(E–H)** and caudal **(I–L)** regions of E12.5 *Pax6* conditional mutant and control embryonic forebrains. Panels **(C,G,K)** show higher power images of regions shown in **(A,E,I)** and panels **(D,H,L)** show higher power images of regions in **(B,F,J)**. Scale bars in **(A,B,E,F,I,J)** = 200 μm; **(C,D,G,H,K,L)** = 20 μm. Pax6cKO = *Emx1-CreER*^T2^;*Pax6*^loxP/loxP^; control = *Emx1-CreER*^T2^;*Pax6*^loxP/+^. Abbreviations: Ctx, cortex; GE, ganglionic eminence, DI, diencephalon. Boxes in **(C,D)** indicate representative areas used for counting *Dll1*+ cells.

As Pax6 is expressed throughout cortical development, from around E8.5, it is possible that our results on the changes in *Dll1* expression in *Pax6*^−/−^ embryos are complicated by the effects of indirect, downstream consequences of the absence of Pax6 activity at earlier stages, including the possibility of compensatory adaptations. We therefore tested the effects of acute, conditional deletion of *Pax6* on cortical expression of *Dll1*. Inducible loss of Pax6 was achieved by combining a tamoxifen-inducible *Emx1-creERT2* transgene that is expressed specifically in cortex (Kessaris et al., 2006) with a conditional (floxed) allele of *Pax6* (Simpson et al., 2009). The genotype of the experimental animals was *Emx1CreEr*^T2^; *Pax6*^loxP/loxP^ (referred to as cKO) and heterozygous littermates (i.e., *Emx1CreEr*^T2^;*Pax6*^loxP/+^) were used as controls. Our previous work with this strain showed that Pax6 protein is still present at E11.5 when tamoxifen is administered at E9.5 (Mi et al., 2013), so we confined our analysis of the cKO strain to E12.5 and E13.5. Both cKO and control mutant embryos expressed *Dll1* in a distinctive salt and pepper pattern at E12.5 and E13.5, similar to that seen in control and *Pax6*^−/−^ embryos (Figure 3).

**Figure 4:**
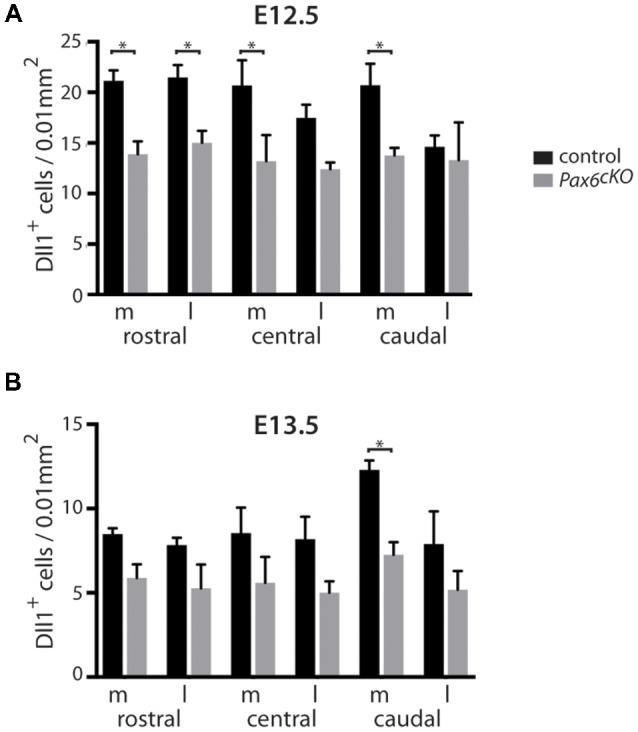
Quantitation of *Dll1+* cells in *Pax*6 c*KO* and control forebrain at E12.5 and E13.5. Bar graphs comparing the density of *Dll1*-expressing cells in specific regions of the cortex in *Pax6* c*KO* mutant and control embryos at E12.5 **(A)** and E13.5 **(B)**. *N* = 3 embryos of each genotype at each age. *p < 0.05, Tukey’s multiple comparison test.

Quantitation revealed a lower mean density of *Dll1*-expressing cells across E12.5 cKO mutant cortex compared to controls (Figure 4A). Two-way ANOVA showed a significant effect of genotype (*p* < 0.0001) and *post hoc* analysis by Tukey’s multiple comparison test confirmed significant decreases in *Dll1*-expressing cell density in cKO cortex at rostromedial (*p* = 0.003), rostrolateral (*p* = 0.01), centromedial (*p* = 0.002) and caudomedial (*p* = 0.005) levels. *Dll1+* cell density was also lower in centrolateral and caudolateral cKO cortex, but the differences were not significant (*p* = 0.08, *p* = 0.99 respectively). Thus, conditional inactivation of *Pax6* caused a widespread significant reduction in the density of *Dll1*-expressing cells in E12.5 cortex.

At E13.5, the mean densities of *Dll1*-expressing cell were lower in cKO cortex than in control embryos (Figure 4B). Two-way ANOVA confirmed a significant effect of genotype (*p* < 0.0001) but *post hoc* analysis (Tukey) indicated that only the caudomedial region of cKO mutant cortex showed a significant reduction in *Dll1* expression at this stage (*p* = 0.012). Overall, conditional mutagenesis had a greater and more widespread effect than constitutive Pax6 loss, suggesting that Pax6 is required during corticogenesis for the production of a normal complement of *Dll1*-expressing cells across cortical regions irrespective of the regional variation in normal levels of Pax6 expression.

### Overexpression of Pax6 has no Effect on Density of *Dll1-*Expressing Cells

**Figure 5:**
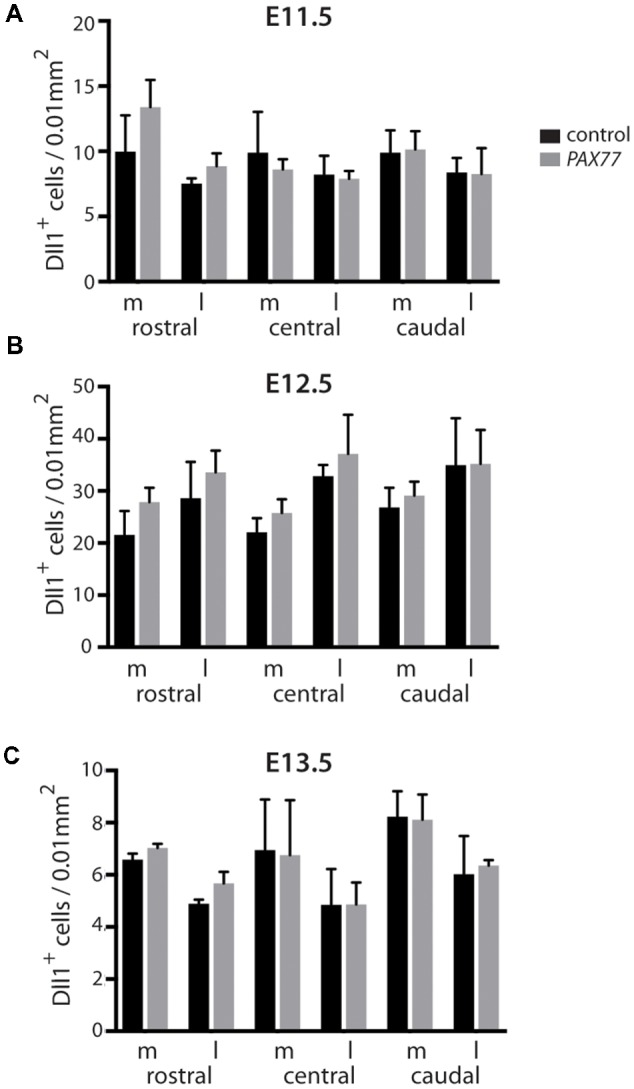
Quantitation of *Dll1*+ cells in *PAX77* and control forebrain at E11.5, E12.5 and E13.5. Bar graphs show the density of *Dll1*-expressing cells in specific regions of the cortex in *PAX77* transgenic and control embryos at E11.5 **(A)**, E12.5 **(B)** and E13.5 **(C)**. No statistically significant changes were found (analysis of variance, ANOVA). *N* = 3 embryos of each genotype at each age.

To further test whether the production of *Dll1*-expressing cells is sensitive to the level of Pax6 expression, we examined embryos from the *PAX77* transgenic mouse strain, which expresses up to three-fold higher levels of PAX6 protein (Manuel et al., 2007). No obvious effect of position or genotype on *Dll1* expression was evident (Figures 5A–C). Two-way ANOVA analysis confirmed no significant effect of genotype at E11.5 or E13.5 (*p* = 0.35, 0.57 respectively) but did indicate an effect of genotype at E12.5 (*p* = 0.008). However, *post hoc* analysis by Tukey’s multiple comparison test showed no significant difference between the density of *Dll1*-expressing cells in *PAX77* and control cortex at any of the six cortical regions examined. This supports the hypothesis that, while Pax6 is required for the production of a normal complement of *Dll1*-expressing cells, its levels do not affect the numbers produced.

### No Apparent Co-expression of *Dll1* and Tbr2 in Developing Cortex

**Figure 6:**
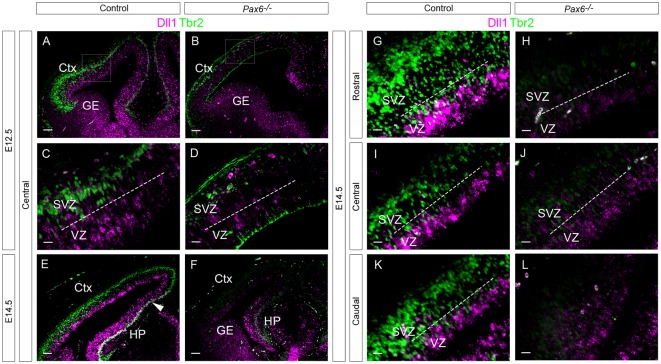
Expression of *Dll1* and Tbr2 in *Pax6*^−/−^ and control embryos at E12.5 and E14.5. *In situ* hybridization for *Dll1* (magenta) and immunohistochemistry for Tbr2 (green) at E12.5 **(A–F)** and E14.5 **(G–L)**. The area delineated by arrowheads in **(E,F)** indicates the presumptive hippocampal region, where *Dll1* and Tbr2 are co-expressed. Dotted lines indicate the boundary between the VZ and the SVZ. Abbreviations: Ctx, cortex; GE, ganglionic eminences; HP, hippocampus; VZ, ventricular zone; SVZ, subventricular zone. Scale bars **(A,B,E,F)**: 80 μm; **(C,D,G–L)**: 20 μm.

Previous studies have shown that loss of Pax6 causes a loss of Tbr2+ IPCs (Quinn et al., 2007). We assessed whether Tbr2+ IPCs express *Dll1* RNA since, if they do, our findings in *Pax6* loss-of-function mutants might be explained by a loss of Tbr2+ IPCs. We combined *in situ* hybridization for *Dll1* with immunohistochemistry for the IPC marker Tbr2 in E12.5 and E14.5 cortex (Figure 6). Strong Tbr2 expression was seen in control embryos at E12.5, spanning the length of the cortex and largely confined to the subventricular zone (SVZ; Figures 6A,C). In contrast, *Dll1* expression was largely restricted to the VZ. We found no evidence of co-expression of *Dll1* and Tbr2 in control cortex at E12.5 (Figures 6A,C), indicating that a clear majority of Tbr2+ cells do not express *Dll1*. As reported previously, the number of Tbr2-expressing cells was greatly reduced in *Pax6*^−/−^ mutants at E12.5 (Figures 6B,D, Quinn et al., 2007). As in controls, no evidence of co-expression of *Dll1* and Tbr2 was found in the *Pax6*^−/−^ cortex (Figures 6B,D).

At E14.5, the SVZ constitutes the majority of the cortical progenitor zone. *Dll1* expression remained largely confined to the narrower VZ region (Figures 6E,G,I,K). As at E12.5, no co-localization between *Dll1* and Tbr2 was observed (Figures 6G,I,K). Interestingly, however, strong co-localization of *Dll1* and Tbr2 was found in the developing hippocampus (HP) region at E14.5 (Figure 6E, region between white arrowheads), indicating that many IPCs in the HP express *Dll1*, in contrast to the neocortex. The presence of *Dll1* expression in both the VZ and SVZ of the developing HP suggests that it is expressed by both RGCs and IPCs in this region.

In E14.5 *Pax6*^−/−^ mutant embryos, we found no cells co-expressing both *Dll1* and Tbr2 in either the cortex or the HP (Figure 6F). As at E12.5, there were many fewer Tbr2-expressing cells in E14.5 *Pax6* ^−/−^ mutants than in controls (Figures 6F,H,J,L).

These findings suggest that the loss of *Dll1*-expressing cells in Pax6 loss-of-function mutants is not a manifestation of the reduction of Tbr2+ IPCs in their cortices. We next considered whether it might be a consequence of altered *Neurog2* expression.

### Overlapping *Neurog2* and *Dll1* Expression in Embryonic Cortex

**Figure 7:**
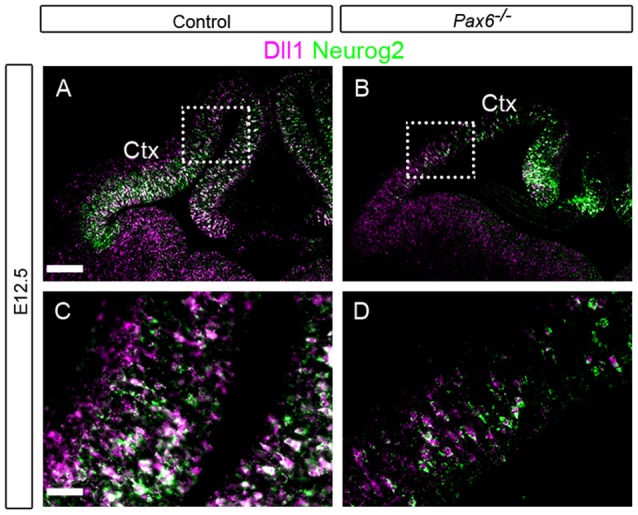
Expression of *Dll1* and *Neurog2* at E12.5 in *Pax6*^−/−^ and control telencephalon. *In situ* hybridizations showing expression of *Dll1* (magenta) and *Neurog2* (green) in control **(A,C)** and *Pax6*^−/−^ **(B,D)** embryos at E12.5. Ctx, Cortex. Scale bars: **(A)** = 100 μm, **(C)** = 25 μm.

As Pax6 can activate expression of *Neurog2* (Scardigli et al., 2003), and Neurog2 can drive expression of *Dll1* (Castro et al., 2006), we tested the possibility that the changes in *Dll1+* cell density found in *Pax6* mutant cortex could be mediated through Neurog2. We first compared the expression of *Neurog2* and *Dll1* in *Pax6*^−/−^ mutants and controls, to test whether cortical progenitor cells express both *Neurog2* and *Dll1*. At E12.5, *Dll1* and* Neurog2* were expressed widely throughout the progenitor zone of the developing cerebral cortex in control embryos and many *Neurog2*/*Dll1* double positive cells were evident (Figures 7A,C) consistent with the possibility that Pax6 could regulate *Dll1* through *Neurog2*. In *Pax6*^−/−^ mutants, there were substantially fewer *Neurog2*-expressing cells, especially in the lateral cortex (Figures 7B,D) and, in contrast to controls, few *Dll*1 N*eurog2* double-positive cells were seen in *Pax6*^−/−^ mutant cortex (Figure 7D), indicating that *Pax6*^−/−^ cells that lacked expression of *Neurog2* were nonetheless able to express *Dll1*.

### *Dll1* Expression Is Unaffected in *Neurog2*^−/−^ Mutant Cortex

**Figure 8:**
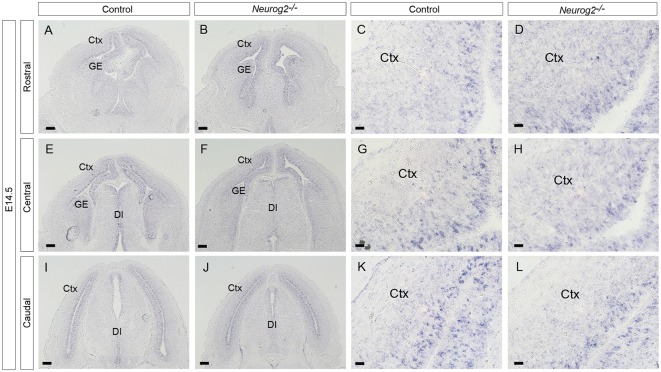
*Dll1* expression in E14.5 *Neurog2*^−/−^ mutant embryos. *In situ* hybridization showing *Dll1* expression in rostral **(A,B)**, middle **(E,F)** and caudal **(I,J)** regions of E14.5 *Neurog2*^−/−^ and control embryonic forebrains. Panels **(C,G,K)** and **(D,H,L)** show higher power images of regions shown in **(A,E,I)** and **(B,F,J)** respectively.

Our hypothesis that Pax6 regulates *Dll1* expression through Neurog2 predicts that decreased expression of Neurog2 should lead to lower *Dll1* expression. However, while *Pax6* mutants showed substantially decreased *Neurog2* expression, the effect on *Dll1* was less pronounced (Figure 7). To test directly whether *Neurog2* regulates *Dll1* expression, we generated homozygous *Neurog2*^−/−^ mutant embryos by intercrossing heterozygous animals carrying a *Neurog2* allele which lacks Neurog2 activity as a result of the insertion of cre recombinase coding sequence (Zirlinger et al., 2002). *Dll1* expression, as shown by *in situ* hybridization, appeared very similar in E14.5 *Neurog2*^−/−^ (Figures 8B,D,F,H,J,L) and control embryos (Figures 8A,C,E,G,I,K), each showing the familiar salt and pepper staining pattern. Quantitation of *Dll1*-expressing cell density in these embryos revealed no statistically significant differences between *Neurog2*^−/−^ mutants and controls (data not shown).

## Discussion

### Evidence for Interactions Between *Pax6* and *Dll1*

To investigate a possible role for the Notch ligand Dll1 in the pathway by which Pax6 influences the balance between proliferation and differentiation of RGC progenitors in embryonic cortex, we characterized the cortical expression of *Dll1* in detail during key neurogenic stages, both in control and *Pax6*^−/−^ mutant embryos. In control embryos at the earliest stages of neurogenesis (E11.5), *Dll1*-expressing cells were significantly more abundant in lateral cortex than medial. Pax6 is expressed in a high lateral to low medial gradient, so the presence of more *Dll1*+ cells in lateral than medial cortex could suggest an effect of Pax6 levels on *Dll1* expression. However, the medial-lateral difference in Dll1 expression was also seen in E11.5 *Pax6*^−/−^ embryos and we found no differences in *Dll1*+ cell density between *Pax6*^−/−^ mutant and control embryos at any position in the E11.5 cortex, arguing against a direct role for Pax6 levels in setting up the medio-lateral difference in *Dll1+* cell density.

At E12.5, the medial-lateral difference in *Dll1* expression was no longer evident. In controls, *Dll1*+ cell density was highest in caudomedial cortex, but this was not significantly different to any other region. In E12.5 *Pax6*^−/−^ mutants, *Dll1* expression was decreased in centrolateral and caudomedial areas of cortex compared to controls, but unaffected in other regions. In E13.5 control embryos, caudomedial cortex again showed the highest density of *Dll1*+ cells, although this was significantly higher than only centrolateral and mediolateral areas. In E13.5 *Pax6*^−/−^ mutants *Dll1+* cells were again significantly reduced in caudomedial cortex, but all other regions showed no significant change in *Dll1*+ cells at this stage. Perhaps surprisingly, the region which showed the most consistent changes in *Dll1+* cells in *Pax6* mutants, caudomedial cortex, is also the region where Pax6 expression levels are lowest.

*Dll1* expression was more strongly affected in conditional *Pax6* mutants. Fewer *Dll1*+ cells were found throughout almost all regions of the E12.5 cortex in cortex-specific *Pax6*^−/−^ mutant embryos, in contrast to constitutive *Pax6*^−/−^ mutants, in which only centrolateral and caudomedial cortex showed significant differences. By E13.5 only caudomedial cortex was affected, the same region affected in constitutive *Pax6*^−/−^ embryos at this stage. A likely explanation for this difference could be the presence of a mechanism which can compensate for the loss of Pax6 in all except caudomedial cortex in constitutive *Pax6*^−/−^ mutants but in the E12.5 cKO mutants has not had enough time after loss of Pax6 for the compensation mechanism to act, such that the effect of Pax6 loss is seen throughout the cortex. The principle that conditional mutants may have more severe phenotypes than constitutive mutants is well established (Rossi et al., 2015). Increasing the level of Pax6 had no significant effect on *Dll1*+ cell numbers, consistent with the finding that gain of Pax6 function generally has a milder effect on cortical development than does loss of function (Manuel et al., 2007; Georgala et al., 2011).

We found large numbers of *Dll1*+ cells in the VZ of mouse embryonic cortex, many of them co-expressing Neurog2, showing that Dll1 is widely expressed in cortical progenitors. However, we did not find any evidence of *Dll1*/Tbr2 co-expressing cells, suggesting that *Dll1* mRNA expression in Tbr2+ basal progenitor cells is less common. Dll1 protein has been detected in Tbr2+ basal progenitors (Nelson et al., 2013), suggesting that the protein persists after Dll1 mRNA is no longer expressed. Indeed, Dll1 protein produced by basal progenitors has been suggested to activate Notch signaling in radial glial progenitors in the VZ (Mizutani et al., 2007; Yoon et al., 2008; Nelson et al., 2013).

### How Might Pax6 Regulate Dll1 Expression?

We found that loss of Pax6 leads to a decrease in the number of *Dll1*+ cells in specific areas of the cortex during neurogenesis. The absence of a straightforward relationship between Pax6 function and expression levels and *Dll1* expression suggests that the interaction between these genes is likely to be indirect. We investigated the possibility that Pax6 could exert its effect on *Dll1* expression indirectly, through the Pax6-regulated neurogenic transcription factor Neurog2 which can regulate *Dll1* (Castro et al., 2006). In support of this possibility, many embryonic cortical progenitor cells express both *Neurog2* and *Dll1* and expression of both of these genes is decreased in *Pax6*^−/−^ mutants. However, no significant difference in *Dll1* expression was observed in *Neurog2*^−/−^ cortex, indicating that the mechanism is not as simple as this. The continued *Dll1* expression in *Neurog2*^−/−^ mutants suggests that the absence of Neurog2 can be compensated for. The most likely candidates to mediate such compensation are the related neurogenic genes *Neurog1* and *Ascl1*. *Neurog1* and *Neurog2* are both normally expressed throughout the cortex, whereas *Ascl1* expression is largely confined to ventral telencephalon (Fode et al., 2000). In *Neurog2*^−/−^ mutants, cortical expression of *Neurog1* is decreased slightly, but *Ascl1* expression is strongly upregulated (Fode et al., 2000). The phenotype of *Ascl1*^−/−^; *Neurog2*^−/−^ double mutants is much more severe than that of *Neurog2*^−/−^ single mutants, indicating that Ascl1 compensates for effects of loss of Neurog2 function (Fode et al., 2000). Further, Ascl1 has been shown to be able to directly activate *Dll1* expression, acting on a different enhancer element to that activated by Neurog2 (Castro et al., 2006). Therefore, increased levels of Ascl1 in *Neurog2*^−/−^ cortex may maintain expression of Dll1 at normal levels. However, *Ascl1* is also upregulated in *Pax6*^−/−^ mutant embryonic cortex (Kroll and O’Leary, 2005), yet significant regional decreases in *Dll1+* cells are observed. This could be consistent with a direct role for Pax6 in regulating Dll1, that cannot be compensated for by Ascl1, or may indicate that the regulation of Dll1 is complex and that other factors are involved.

## Data Availability

All datasets generated for this study are included in the manuscript.

## Author Contributions

ED designed and performed experiments and data analysis. ED, DP and JM discussed experimental design and results and wrote the manuscript. All authors read and approved the final manuscript.

## Conflict of Interest Statement

The authors declare that the research was conducted in the absence of any commercial or financial relationships that could be construed as a potential conflict of interest.
